# A focus on the use of real-world datasets for yield prediction

**DOI:** 10.1039/d3sc90069j

**Published:** 2023-04-27

**Authors:** Latimah Bustillo, Tiago Rodrigues

**Affiliations:** a Research Institute for Medicines (iMed), Faculty of Pharmacy, University of Lisbon Av Prof Gama Pinto 1649-003 Lisbon Portugal tiago.rodrigues@ff.ulisboa.pt

## Abstract

The prediction of reaction yields remains a challenging task for machine learning (ML), given the vast search spaces and absence of robust training data. Wiest, Chawla *et al.* (https://doi.org/10.1039/D2SC06041H) show that a deep learning algorithm performs well on high-throughput experimentation data but surprisingly poorly on real-world, historical data from a pharmaceutical company. The result suggests that there is considerable room for improvement when coupling ML to electronic laboratory notebook data.

## Abstract 2

A machine learning workflow is used to predict reaction yields using data in a corporate electronic laboratory notebook.

## Graphical abstract

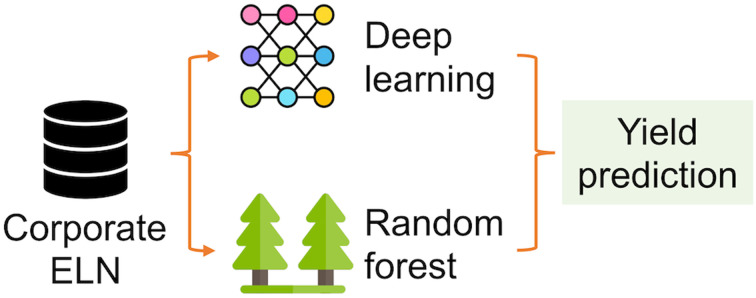

Machine learning (ML) has seen formidable applications in diverse fields of science, including chemistry.^1^ The prediction of retrosynthetic routes,^2^ the *de novo* design of chemical entities,^3^ and the prediction of pharmacological profiles for small molecules^4^ are just a few examples where ML is currently making an impact and accelerating research.^5,6^ These advances are made possible chiefly due to improved algorithms, methods for describing molecular structure and, above all, available datasets. In fact, a corollary in ML research is that a model can only be as good as its training data.^7^ To that end, a considerable amount of time in the ML development process is devoted to collecting, curating and harmonizing data.

With the emergence of large language models (LLMs; *e.g.*, ChatGPT) that ‘converse’ with human users one may assume that predicting reaction yields is only a simple task. That is not the case. Predicting yields remains challenging due to the absence of robust reaction precedents.^8^ Among the many shortcomings, datasets tend to be biased towards productive reactions. This has deep roots in the chemistry literature given the preference to report positive results and omit ‘failures’.^9^ It is also known that observational uncertainty affects the reported yield (usually measured once rather than verified through replicates) and, in some cases, the values are misassigned in databases.^5^ These errors propagate and impact the ML model accuracy. Finally, the search spaces are so vast that any currently available dataset remains sparse in terms of coverage. As a result, there is a need to develop new ML methodologies and approaches that mitigate the referred limitations.

Active and reinforcement learning have been previously employed in the optimization of reaction conditions using yield as a metric to gauge success.^10,11^ High-throughput experimentation (HTE) data is a viable starting point for reaction optimization campaigns and is compatible with diverse flavours of molecular descriptors.^8,10,12^ Those data can however be too focused on certain regions of the search space and the resulting ML tools might generalize poorly. It has been hypothesized that electronic laboratory notebooks (ELNs) – for example those from pharmaceutical companies – can provide less biased dataset alternatives to HTE datasets. As an additional selling point, the larger chemical space coverage in ELNs may be instrumental for improved model generalizability and the identification of new reactivity patterns. Still, the utility of ELNs as data sources remained unknown until now partly due to their restricted access.

In a collaborative study,^13^ Wiest (Notre Dame), Chawla (Notre Dame), Doyle (University of California), Norrby (AstraZeneca) and co-workers delved into ELNs to build ML models and predict reaction yields. Using pharmaceutically relevant transformations as examples (*e.g.*, Buchwald–Hartwig and Suzuki couplings) it was found that state-of-the-art representation learning performs unsatisfactorily and not much better than other simpler methods, such as random forests.

The team started by querying AstraZeneca's legacy data and pre-processing the retrieved information. As expected, incomplete reactions and those with 0% yield were highly prevalent in the ELN. Still, a total of 781 Buchwald–Hartwig reactions fulfilled the pre-established quality criteria, which was a minute number of reactions in comparison to the whole search space size (∼4.7 × 10^8^). Notwithstanding the diversity of the ELN data, its size was in stark contrast with a related HTE dataset which comprised 3960 Buchwald–Hartwig reactions covering a space of 4140 possibilities for only five distinct products. These datasets were then used to train ML models with chemically meaningful descriptors. The performance of random forests, BERT, *k*-nearest neighbours, Lasso, and support vector machines was subsequently compared. For a realistic assessment of the expected baseline performance, Y-randomized (shuffled) datasets were also generated and optimized models were trained. From all the surveyed methods, the random forests and BERT models performed best for HTE datasets, providing *r*^2^ values > 0.82.

In comparison, the ELN data did not afford a model with satisfactory performance. Random forests performed best but with an *r*^2^ value of only 0.266. While the obtained performance was slightly better than a randomized baseline, the result suggested that the vast search space was only sparsely populated by the training data. As a potential solution, the authors hypothesized that the maximum amount of information could be captured by retaining elements of the two best performing methods in HTE datasets – random forests and BERT. This meant that an optimized model – herein named YieldGNN – would combine molecular properties (*i.e.*, chemical features or descriptors) from the tabular data used in random forests with the molecular graph structure in attributed graph neural networks (GNN; Fig. 1).

**Fig. 1:**
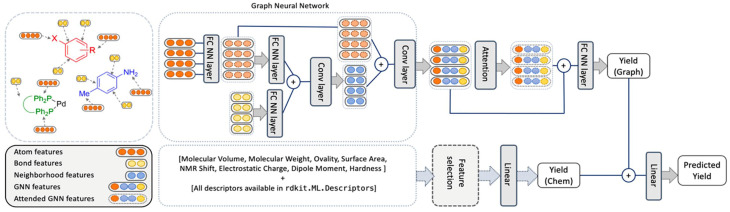
Architecture of the YieldGNN method that combines structural (atom and bond features over their neighbourhood) with chemical features stemming from a random forest. The two yield scores that originate from the graph-based and ensemble tree methods are concatenated and passed through a linear layer to generate the final predicted yield. Figure reproduced from Wiest, Chawla *et al.* (https://doi.org/10.1039/D2SC06041H).

In short, Weisfeiler–Lehman networks were employed wherein atom and bond information was aggregated using convolutional operations. The embedded neighbourhood features were then concatenated with atom representations to ultimately access attended GNN features that were the basis of yield predictions. To avoid overfitting of the neural network, a random forest was trained in parallel, and its outputs were linearly combined to those of the attended GNN to afford a final yield prediction. The resulting composite model outperformed all previous methods on HTE datasets, including the random forests, as indicated by more favourable *r*^2^ and mean absolute error values. However, as the authors correctly mention, the obtained improvements are more relevant from a statistical point of view than in a real-life laboratory setting. This owes to the fact that the marginally higher *r*^2^ value absorbs uncertainties and errors in yield measurements. Still, the results show some evidence that connectivity data is important for model performance and that its inclusion is a viable strategy for future ML implementations.

The application of YieldGNN to ELN data provided less promising and unexpected results in comparison. No meaningful predictions were obtained (*r*^2^ ≤ 0) even after extensively fine-tuning the base model. One of the reasons for this observation was the inability of YieldGNN to learn key features that govern the transformation, according to weight values that did not surpass 0.05.

In an attempt to solve this issue, the research team investigated whether pre-training of a GNN with a large corpus of molecules could help improving the ML model performance. Three different pre-training approaches were surveyed, including attribute masking, context or edge prediction, followed by transfer learning with the relevant datasets for yield prediction. Rather surprisingly, those GNN models performed worse than the random forest baseline for HTE data, likely due to a domain mismatch. A marginal improvement was observed for the corporate data (*r*^2^ = 0.177 *vs. r*^2^ = 0.049), but the ML utility remained far from ideal for prospective applications. Altogether, the obtained results suggest that future ML implementations should exploit datasets that are closer to the problem of interest. Despite our ability to generate large volumes of data, this study also shows that the underlying experimental design rarely has a ML application in mind – *i.e.*, ‘big data’ is not linearly linked to information and model performance. Further, YieldGNN is conceptually different from the previously reported random forest^10^ and Bayesian optimization^8^ approaches for yield optimization that use reaction conditions and/or density functional descriptors. Together with the implementation of LLM chemistry agents^14,15^ to streamline experimental work, the future of data-driven chemistry and yield prediction in particular does seem promising. Still, we re-emphasize a recent call for metrics in ML model evaluation that go beyond accuracy.^16^ Said metrics should gauge advances with sustainability in mind. In summary, Wiest and colleagues provide an excellent account on ML models for yield prediction, the importance of information (arguably higher than the importance of the algorithm) and hint at a clear disconnect between ML performance and the superiority of corporate ELNs over publicly available data sources. Whether those conclusions generalize to other ELNs remains to be seen.

## Author contributions

L. B. and T. R. wrote the manuscript.

## Conflicts of interest

T. Rodrigues is a co-founder and shareholder of TargTex SA, and a consultant to the pharmaceutical industry.

## Supplementary Material
